# Ratiometric Fluorescence Detection of Colorectal Cancer-Associated Exosomal miR-92a-3p with DSN-Assisted Signal Amplification by a MWCNTs@Au NCs Nanoplatform

**DOI:** 10.3390/bios12070533

**Published:** 2022-07-17

**Authors:** Zhiwei Sun, Juan Li, Yao Tong, Li Zhao, Xiaoyu Zhou, Hui Li, Chuanxin Wang, Lutao Du, Yanyan Jiang

**Affiliations:** 1 Key Laboratory for Liquid−Solid Structural Evolution and Processing of Materials, Ministry of Education, Shandong University, Jinan 250061, China; sun_zhiwei@mail.sdu.edu.cn (Z.S.); 202020473@mail.sdu.edu.cn (L.Z.); zhouxiaoyu322@163.com (X.Z.); lihuilmy@hotmail.com (H.L.); 2 Shenzhen Research Institute of Shandong University, Shenzhen 518057, China; 3 Department of Clinical Laboratory, The Second Hospital, Cheeloo College of Medicine, Shandong University, Jinan 250033, China; niderouke@163.com (J.L.); tooong1997@126.com (Y.T.); cxwang@sdu.edu.cn (C.W.); 4 Shandong Engineering & Technology Research Center for Tumor Marker Detection, Jinan 250033, China; 5 Shandong Provincial Clinical Medicine Research Center for Clinical Laboratory, Jinan 250033, China

**Keywords:** multi-walled carbon nanotubes, Au nanoclusters, biosensor, ratiometric fluorescence, duplex-specific nuclease, exosomal miRNA

## Abstract

The detection of miRNA shows great promise in disease diagnosis. In this work, a ratiometric fluorescent biosensor based on multi-walled carbon nanotubes@gold nanoclusters (MWCNTs@Au NCs) and duplex-specific nuclease (DSN)-assisted signal amplification was fabricated for miRNA detection. Colorectal cancer (CRC)-associated miR-92a-3p extracted from exosomes was selected as the target. MWCNTs@Au NCs performs the dual functions of fluorescence quencher and internal fluorescence reference. In the absence of miR-92a-3p, an Atto-425-modified single-stranded DNA probe is adsorbed on MWCNTs@Au NCs, resulting in the quenching of Atto-425. In the presence of miR-92a-3p, the duplex is formed by hybridization of the probe and miR-92a-3p and leaves the MWCNTs@Au NCs, resulting in the fluorescence recovery of Atto-425. DSN can cleave the probe and result in the release of miR-92a-3p. The released miR-92a-3p can hybridize with other probes to form a signal amplification cycle. The fluorescence of MWCNTs@Au NCs remains stable and constitutes a ratiometric fluorescence system with that of Atto-425. A detection concentration interval of 0.1–10 pM and a limit of detection of 31 fM was obtained under optimized measurement conditions. In addition, the accuracy of the biosensor was validated by detecting the concentration of miR-92a-3p extracted from clinical exosome samples.

## 1. Introduction

MicroRNAs (miRNAs) are small, single-stranded, noncoding RNAs of 18–24 nucleotides that play key roles in many biological processes, including cell proliferation, differentiation, stress resistance and apoptosis by regulating gene expression at the post-transcriptional level [1,2,3]. Numerous studies have found that changes in miRNA expression are closely related to the occurrence of various diseases, such as cardiovascular disease, obesity, diabetes and tumors [4,5,6,7]. Therefore, detection of the expression level of miRNAs in organisms can provide valuable information for biomedical research and early disease diagnosis [8,9]. Based on this, the exploitation of fast, sensitive and selective sensing strategies for miRNAs has attracted immense interest.

Effective detection of miRNAs is challenging due to their small size, low expression level and high sequence similarity [10,11]. Recently, several signal amplification strategies, including real-time quantitative polymerase chain reaction (RT-qPCR), hybridization chain reaction, rolling circle amplification, nicking enzyme and duplex-specific nuclease (DSN), have been developed to improve the detection sensitivity for miRNA [12,13,14,15,16]. Further, miRNA biosensing based on DSN-assisted signal amplification is considered a promising strategy. DSN-amplified miRNA detection has been known for a decade, and since then there have been many examples reported [17]. DSN can cleave DNA in DNA–RNA heteroduplexes into DNA fragments while keeping the RNA strand intact, which indicates that the complete miRNA target can be reused to form a new heteroduplex structure for the next step of cleavage [18,19]. DSN cleavage of those duplexes with more than 10–12 base complementary pairs ensures the high selectivity for the target [20]. Meanwhile, fluorescence-based detection methods are commonly used in bioassays because of their excellent sensitivity and rapid analysis [21,22]. Based on the above merits, the combination of DSN-assisted target cyclic signal amplification and fluorescence-based detection can achieve sensitive, selective and fast detection of miRNA.

Fluorescence-quenching nanomaterials such as Mxenes, graphene oxide, g-C_3_N_4_, MoS_2_, gold nanoparticles (Au NPs) and multi-walled carbon nanotubes (MWCNTs) have been widely used in biosensing [23,24,25,26,27,28]. Among them, MWCNTs have a large adsorption surface and can act as an excellent substrate for the preparation of MWCNT-based nanocomposites [29,30,31]. For instance, in the work of Ma et al., MWCNTs were used as an excellent fluorescence quenching platform for the detection of miRNA-155 in human serum [30]. The synthesized MWCNT/Au NPs showed a quenching efficiency of 93.2% for a FAM-labeled single-stranded DNA probe, paving the way for the sensitive detection of miRNA-155. However, the reliability of this detection strategy, which relies on a single fluorophore, is easily disturbed by unstable factors such as the light source and complex solution environment [32,33]. To overcome this limitation, ratiometric fluorescence biosensing has received increasing attention due to its capability to counteract the effects of instabilities [34,35,36].

In this work, we successfully synthesized multi-walled carbon nanotubes@gold nanoclusters (MWCNTs@Au NCs) with dual functions of fluorescence emission and quenching by linking Au NCs to MWCNTs through Au–S bonds. A sensitive and selective ratiometric fluorescent biosensor based on MWCNTs@Au NCs and DSN-assisted signal amplification was fabricated for miRNA detection. Colorectal cancer (CRC)-associated miR-92a-3p extracted from exosomes served as the target in view of its high abundance in exosomes [37]. The morphology, surface properties and optical characteristics of MWCNTs@Au NCs were firstly analyzed. Then, the fluorescence signals of the biosensing system were measured, and the feasibility of biosensing was verified. Subsequently, the measurement conditions of the biosensor were optimized, and the sensitivity and selectivity of detection were explored. Finally, we validated the accuracy of the proposed biosensor in the detection of miR-92a-3p extracted from exosomes by RT-qPCR.

## 2. Materials and Methods

### 2.1. Samples and Materials

All serum samples were obtained from healthy controls and CRC patients at the Second Hospital of Shandong University. This work was approved by the ethics committee of the Second Hospital of Shandong University.

Tetrachloroauric(III) acid tetrahydrate (HAuCl_4_·4H_2_O, AR grade), glutathione reduced (C_10_H_17_N_3_O_6_S, ≥98%), β-mercaptoethylamine (C_2_H_7_NS, 98%), concentrated sulfuric acid (H_2_SO_4_, GR grade) and concentrated nitric acid (HNO_3_, AR grade) were purchased from Sinopharm Chemical Reagent Co., Ltd. Multi-wall carbon nanotubes (ID: 5–10 nm, OD: 30–50 nm, length: <10 μm), N-(3-dimethylaminopropyl)-N’-ethylcarbodiimide hydrochloride (EDC; C_8_H_18_ClN_3_, 98.5%), N-hydroxysuccinimide (NHS; C_4_H_5_NO_3_, 99%), Tris-HCl buffer (1 M, pH 7.4) and MES buffer (0.5 M, pH 5.5) were purchased from Shanghai Macklin Biochemical Co., Ltd. (Shanghai, China). DSN was purchased from Evrogen JSC. The probe was purchased from Sangon Biotech (Shanghai) Co., Ltd. (Shanghai, China). The miR-92a-3p and mismatched sequences were purchased from Shanghai Genepharma Co., Ltd. (Shanghai, China). Ultrapure water was used in all experiments. All oligonucleotide sequences are listed in Table 1.

### 2.2. Synthesis of Fluorescent Au NCs

Firstly, 10 mL tetrachloroauric(III) acid tetrahydrate aqueous solution (4 mM) was added to a three-necked flask and stirred at 25 °C for 5 min under a nitrogen atmosphere. Then, 10 mL glutathione reduced aqueous solution (6 mM) was injected and stirred for 10 min. Subsequently, the solution was refluxed at 100 °C for 12 h. The purified Au NCs aqueous solution was obtained by dialysis with ultrapure water for 24 h.

### 2.3. Preparation of MWCNTs@Au NCs

For carboxylation of MWCNTs [38], MWCNTs (0.1 g) were added to 20 mL of a mixture of concentrated sulfuric acid and concentrated nitric acid (3:1 by volume) and sonicated for 4 h. Then, the mixture was centrifuged and washed repeatedly with ultrapure water and dried at 60 °C for 12 h to obtain carboxylated MWCNTs.

For conjugation of Au NCs and MWCNTs, MWCNTs (10 mg), MES buffer (20 μL; 0.5 M, pH 5.5), EDC (10 mg) and NHS (10 mg) were dispersed in 10 mL ultrapure water and stirred at 30 °C for 0.5 h to activate the carboxyl of the MWCNTs. Next, Tris-HCl buffer (200 μL; 1 M, pH 7.4) and β-mercaptoethylamine (10 mg) were added and stirred for 12 h. Subsequently, the solution was dialyzed with ultrapure water for 24 h to obtain thiol-modified MWCNTs. Finally, 10 mL Au NCs aqueous solution (0.39 mg/mL) was added to the above solution and stirred for 24 h to obtain MWCNTs@Au NCs. The concentration of MWCNTs@Au NCs was calculated to be 0.7 mg/mL by weighing the mass of the MWCNTs@Au NCs collected by freeze-drying.

### 2.4. Fluorescence Detection of miR-92a-3p

Probe (1 μL, 10 μM) and Tris-HCl buffer (10 μL; 1 M, pH 7.4) were added to MWCNTs@Au NCs solution (100 μL, 0.7 mg/mL) and maintained at 25 °C for 40 min. Then, the target (5 μL) with different concentrations, DSN master buffer (10 μL, used as obtained) and DSN (0.2 μL) were added and incubated at 45 °C for 2 h. After that, DSN stop solution (5 μL, 10 mM EDTA) was added and kept for 10 min to inactivate DSN. The solution volume was quantified to 2 mL by adding ultrapure water. The fluorescence intensities were collected using a fluorescence spectrophotometer. The excitation wavelength was 430 nm, and the slits for both excitation and emission were set to be 15 nm. The fluorescence spectra in the range of 465–700 nm were collected. All measurements were performed in parallel three times, and the averages were reported.

### 2.5. Extraction of Exosomes and Exosomal RNAs

ExoQuickTM Exosome Precipitation Solution (System Biosciences, Los Angeles, CA, USA) was used to extract exosomes from 0.3 mL serum according to the manufacturer’s protocols. The marker proteins CD63 and CD9 of exosomes were analyzed by Western blotting. Total RNAs of exosomes were extracted by miRNeasy Micro Kit (50) (Qiagen, Dusseldorf, Germany) according to the manufacturer’s instruction. Total RNA solution was quantified to 30 μL by adding ultrapure water.

### 2.6. Characterization

The morphology of MWCNTs, MWCNTs@Au NCs and exosomes were observed by a transmission electron microscope (TEM; HT7700, Hitachi). The zeta potentials of MWCNTs and MWCNTs@Au NCs were measured by a zeta potential analyzer (Zetasizer NanoZS, Malvern). The Fourier transform infrared spectra (FTIR) of MWCNTs and MWCNTs@Au NCs were collected by a Fourier transform infrared spectrometer (Tensor 37, Bruker). The fluorescence properties of MWCNTs@Au NCs were measured by a fluorescence spectrophotometer (RF-6000, Shimadzu). The ultraviolet–visible (UV–vis) absorption spectra of MWCNTs and MWCNTs@Au NCs were collected by an ultraviolet–visible spectrophotometer (Specord 200 plus, Analytikjena).

### 2.7. RT-qPCR Detection of miR-92a-3p

RT-qPCR was used as the reference to test the accuracy of the ratiometric fluorescent biosensor. A total of 3.75 μL of RNAs were reverse transcribed by Mir-X™ miRNA First-Strand Synthesis (Takara, Kusatsu, Japan) on T100TM Thermal Cycler (Bio-Rad, Shanghai, China) to synthesize cDNAs. RT-qPCR was carried out by SYBR^®^ Green Premix Pro Taq HS qPCR Kit AG11701 (Accurate Biology, Shanghai, China) on a CFX96TM Real-Time System (Bio-Rad, China). The calibration line was drawn based on the real-time fluorescence curves obtained at different concentrations of miR-92a-3p.

## 3. Results and Discussion

### 3.1. Characterization of Materials

**Figure 1:**
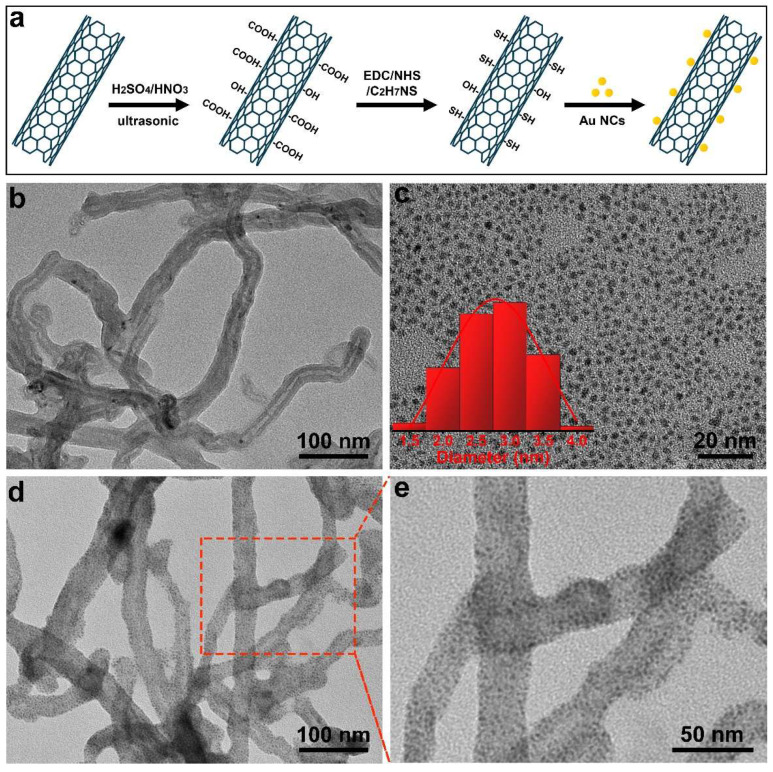
(**a**) Schematic illustration of the synthesis of MWCNTs@Au NCs. TEM images of (**b**) MWCNTs, (**c**) Au NCs (the inset depicts the size distribution of Au NCs) and (**d**,**e**) MWCNTs@Au NCs.

MWCNTs@Au NCs were synthesized following the procedure illustrated in Figure 1a. First, MWCNTs were carboxylated by concentrated sulfuric acid and concentrated nitric acid according to Equation (1), and the water solubility of the MWCNTs was improved by carboxylation [39]. Then, the carboxyl groups of the MWCNTs were activated by EDC and NHS, and the sulfhydryl groups were modified onto MWCNTs by amidation of the carboxyl groups with the amino groups of the β-mercaptoethylamine. Finally, the sulfhydryl groups of MWCNTs and Au NCs formed Au–S bonds via incubation. The morphologies of MWCNTs, Au NCs and MWCNTs@Au NCs were observed by TEM. As shown in Figure 1b, the hollow MWCNTs display outer diameters of 30–50 nm and are well dispersed in water. The prepared Au NCs with glutathione as a reducing agent show a size range of 2–3.5 nm and good monodispersity in water (Figure 1c). For the preparation of MWCNTs@Au NCs, thiol was firstly modified on the carboxylated MWCNTs by amidation. Then, Au–S bonds were formed between MWCNTs and Au NCs by adding Au NCs and reacting for 24 h. The TEM image of MWCNTs@Au NCs demonstrates that dense Au NCs are uniformly distributed on MWCNTs (Figure 1d,e). The absence of monodispersed Au NCs in MWCNTs@Au NCs solution indicates that the Au NCs are fully conjugated to MWCNTs.

(1) $$\mathrm{MWCNT}+{\mathrm{HNO}}_{3}\;\overset{H^{+}}{\to }\;\mathrm{MWCNT}-{\mathrm{COO}}^{-}+{\mathrm{NO}}_{2}+H_{2}O\;\to \;\mathrm{MWCNT}-\mathrm{COOH}$$

**Figure 2:**
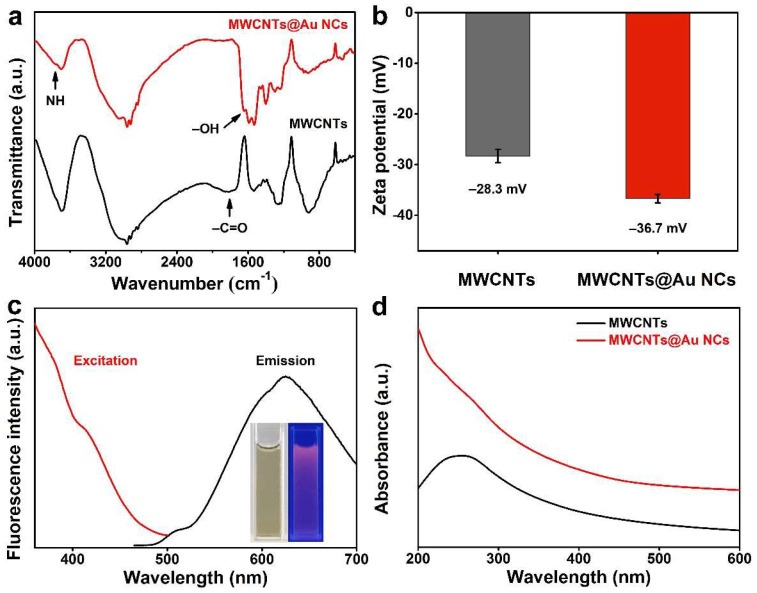
(**a**) FTIR spectra and (**b**) zeta potentials of MWCNTs and MWCNTs@Au NCs. (**c**) Fluorescence excitation and emission spectra of MWCNTs@Au NCs (the inset shows the optical photographs of MWCNTs@Au NCs under sunlight (left) and 360 nm UV light (right)). (**d**) UV–vis absorption spectra of MWCNTs and MWCNTs@Au NCs.

Conjugation between MWCNTs and Au NCs was further confirmed by FT-IR analysis. As can be seen from Figure 2a, a distinct characteristic band around 1710 cm^−1^ attests to carboxyl existing in the spectrum of MWCNTs, implying that the MWCNTs are rich in carboxyl. In the spectrum of MWCNTs@Au NCs, a characteristic band at 3765 cm^−1^ attributed to amide appears, while that of carboxyl disappears, indicating that the β-mercaptoethylamine has been modified onto MWCNTs. In addition, a characteristic band at 1645 cm^−1^ appeared in the spectrum of MWCNTs@Au NCs, demonstrating the successful conjugation of MWCNTs and Au NCs [40]. The zeta potential of MWCNTs is -28.3 mV due to its surface rich in carboxyl and hydroxyl (Figure 2b). This means that MWCNTs have excellent water dispersibility. MWCNTs@Au NCs shows more negative zeta potential of -36.7 eV, indicating that it has better water dispersibility than MWCNTs. Despite the fluorescence-quenching performance of MWCNTs, the MWCNTs@Au NCs still emit intense red fluorescence under excitation at 360 nm, as depicted in the inset of Figure 2c. The fluorescence emission peak of MWCNTs@Au NCs is located at 625 nm, and its fluorescence intensity decreases with increasing excitation wavelength. In the UV–vis absorption spectrum of MWCNTs, there is a typical carbon skeleton absorption peak located at 200–300 nm (Figure 2d). The absorbance of MWCNTs@Au NCs decreases with the increase of irradiation wavelength, which is attributed to the weakening of the plasmonic resonance of Au NCs [41].

### 3.2. Detection Principle of miR-92a-3p

**Figure 3:**
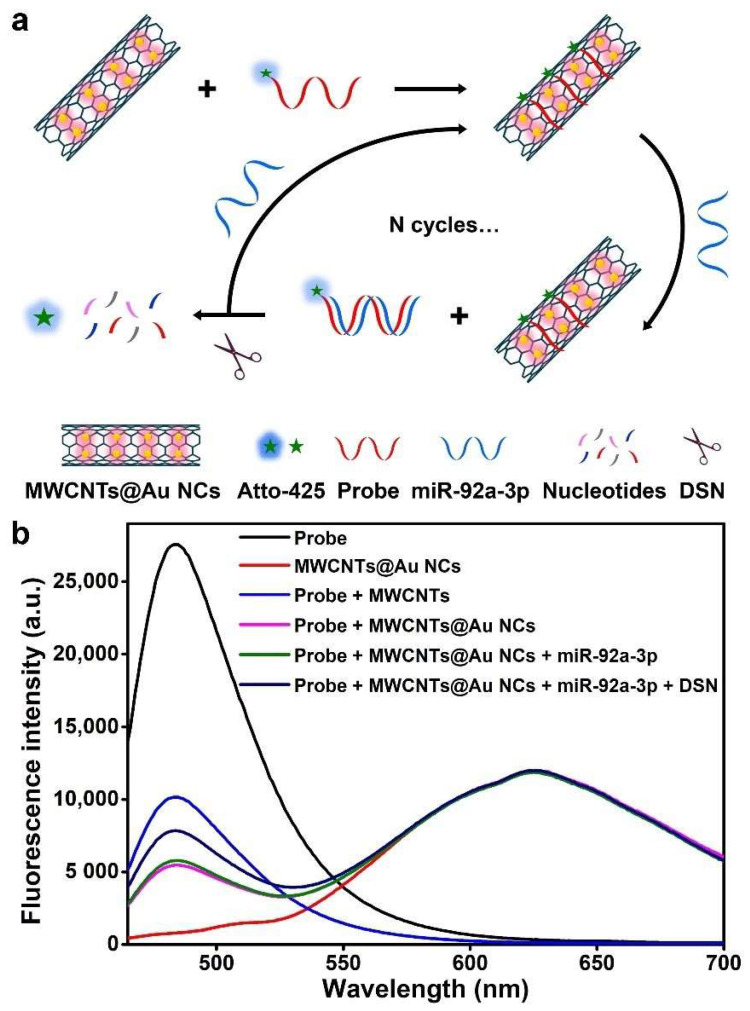
(**a**) Schematic diagram of fluorescence detection of miR-92a-3p based on MWCNTs@Au NCs and DSN-assisted signal amplification. (**b**) Fluorescence spectra of probe, MWCNTs@Au NCs, probe + MWCNTs, probe + MWCNTs@Au NCs, probe + MWCNTs@Au NCs + miR-92a-3p, and probe + MWCNTs@Au NCs + miR-92a-3p + DSN.

As depicted in Figure 3a, in the absence of miR-92a-3p, single-stranded DNA probes are adsorbed on the surface of MWCNTs@Au NCs, and the fluorescence of Atto-425 is quenched. When miR-92a-3p appears, the probe–miR-92a-3p hybrid duplex is formed and leaves from MWCNTs@Au NCs. The fluorescence of Atto-425 is thus recovered. Then, the probe in the duplex is cleaved by DSN and subsequently miR-92a-3p is released to participate in the next hybridization to form a signal amplification cycle. Therefore, the fluorescence intensity of Atto-425 is positively correlated with the concentration of miR-92a-3p, while the fluorescence intensity of Au NCs remains stable. In theory, the concentration of miR-92a-3p is positively correlated with the ΔAtto-425/Au NCs fluorescence intensity ratio.

The fluorescence signals of different material systems were measured to evaluate the feasibility of the biosensing (Figure 3b). The fluorescence peak of Atto-425 is located at 482 nm and is 143 nm away from that of Au NCs, which avoids interference of the two fluorescent materials with each other. MWCNTs@Au NCs show stronger fluorescence quenching than MWCNTs, which is attributed to the enhanced effect of the fluorescence quenching capability of Au NCs [42,43]. The addition of 1 pM miR-92a-3p results in a slight recovery of the fluorescence of Atto-425, which is attributed to the formation of the probe–miR-92a-3p hybrid duplex. The addition of DSN results in more pronounced fluorescence recovery of Atto-425 in the presence of 1 pM miR-92a-3p, demonstrating the feasibility of the DSN-assisted signal amplification strategy. Therefore, the proposed ratiometric fluorescent biosensor can be used for sensitive detection of miR-92a-3p.

### 3.3. Optimization of Measurement Conditions

**Figure 4:**
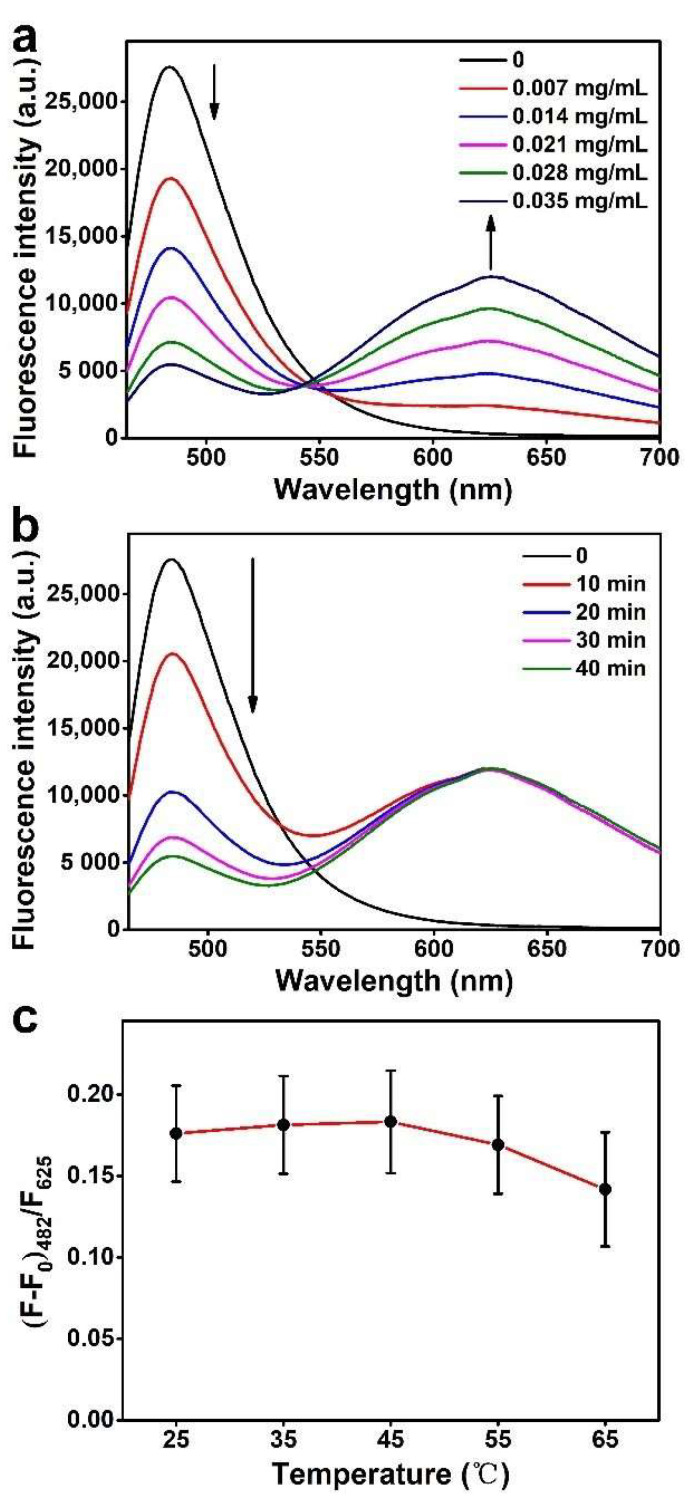
(**a**) The effect of the amount of MWCNTs@Au NCs on the fluorescence signals of the biosensing system. (**b**) The variation of the fluorescence of Atto-425 with the mixing time of the probe and MWCNTs@Au NCs. (**c**) The effect of incubation temperature on the fluorescence signals (*n* = 3, mean ± s.d.).

The effect of the amount of MWCNTs@Au NCs on the fluorescence signals of the biosensing system was investigated. As shown in Figure 4a, the fluorescence of Atto-425 decreases with the increase of MWCNTs@Au NCs. When the amount of MWCNTs@Au NCs is increased from 0.028 mg/mL to 0.035 mg/mL, the decrease in the fluorescence intensity of Atto-425 slows down. So, 0.035 mg/mL MWCNTs@Au NCs aqueous solution is determined as optimal. Variation of the fluorescence intensity of Atto-425 with the mixing time of the probe and MWCNTs@Au NCs was investigated to determine the time required to achieve stable adsorption and quenching (Figure 4b). Within 10 min of mixing, the fluorescence of Atto-425 decreases significantly. The fluorescence intensity of Atto-425 decreases sharply at 10–20 min, indicating that a large number of probes are adsorbed on the surface of MWCNTs@Au NCs. A slight decrease in the fluorescence intensity is observed at 30–40 min, indicating that most of the probes are adsorbed on the surface of MWCNTs@Au NCs within 40 min. Therefore, 40 min is selected as the optimal mixing time. The activity of DSN and hybridization between complementary single-stranded nucleic acids are significantly affected by the incubation temperature [41]. Therefore, the effect of incubation temperature on the fluorescence signals of the biosensing system at a miR-92a-3p concentration of 1 pM was investigated. As shown in Figure 4c, the maximum (F-F_0_)482/F625 value is obtained at 45 °C, while a lower or higher temperature results in a decreased (F-F_0_)482/F625 value due to inefficient hybridization between probe and miR-92a-3p or reduction in the activity of DSN [44]. Thus, 45 °C is determined to be the optimal incubation temperature.

### 3.4. Sensitivity and Selectivity for miR-92a-3p Detection

**Figure 5:**
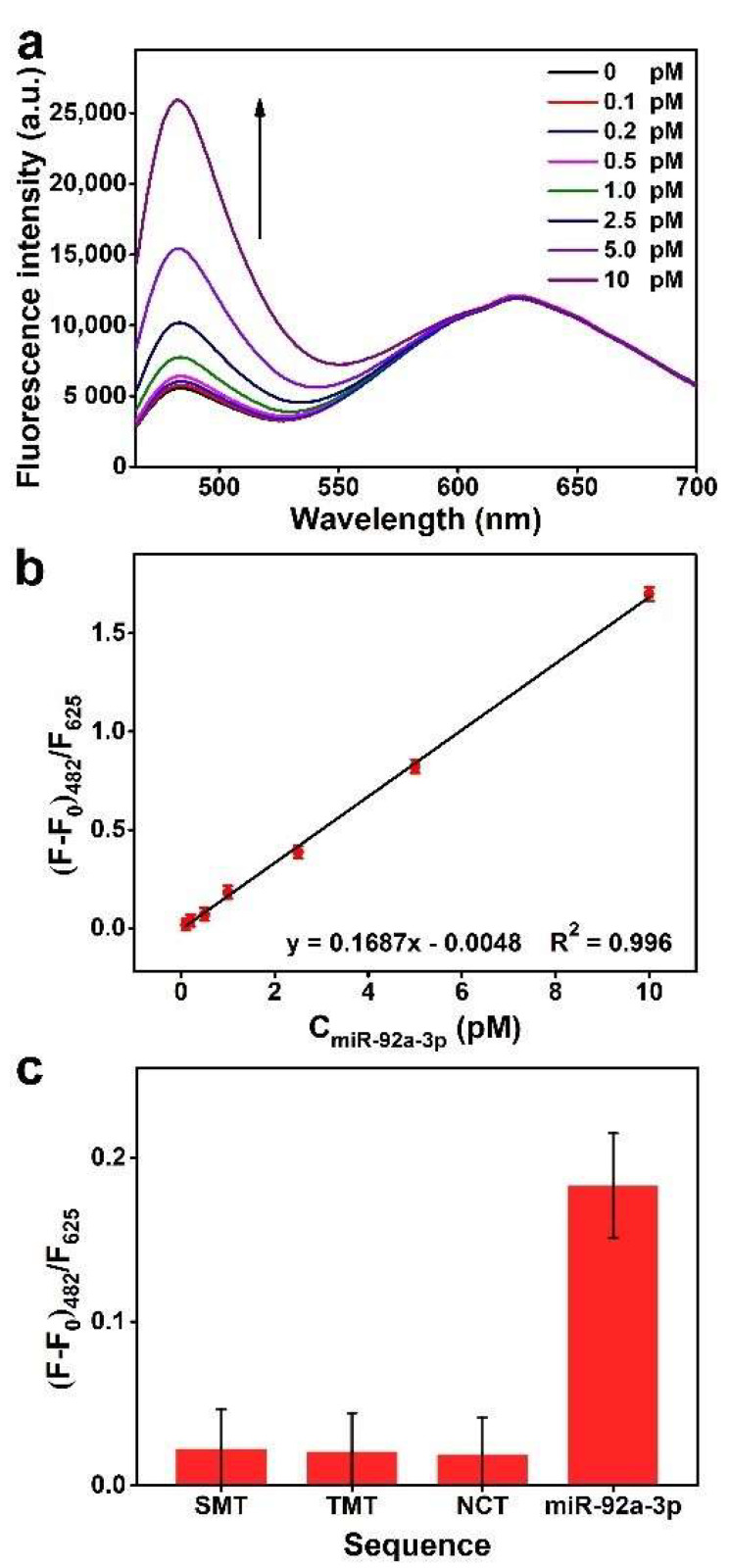
(**a**) Fluorescence spectra of the biosensor under different concentrations of miR-92a-3p. (**b**) The calibration line of the Δprobe/MWCNTs@Au NCs fluorescence ratio against the concentration of miR-92a-3p (*n* = 3, mean ± s.d.). (**c**) Comparison of the fluorescence signals produced by miR-92a-3p and mismatched sequences (*n* = 3, mean ± s.d.).

The sensitivity of the proposed biosensor to miR-92a-3p was measured under optimized conditions. Figure 5a–b show that the fluorescence intensity of Atto-425 linearly increases along with the increase of miR-92a-3p concentration from 0.1 to 10 pM, and a calculated detection limit of 31 fM is obtained based on 3σ. The goodness-of-fit of the regression equation is 0.996, indicating that the biosensor shows excellent regularity and stability. Compared to previously reported ratiometric fluorescent biosensors and DSN signal amplification-based fluorescent biosensors enumerated in Table 2, our fabricated biosensor exhibits a satisfactory linear detection interval and detection limit, demonstrating its potential for sensitive detection of miRNAs. In addition, to evaluate detection selectivity, concentration assays were performed with SMT, TMT and NCT as control targets. The preset concentration of all sequences was 1 pM, and the fluorescence signals they produced are presented in Figure 5c. The (F-F_0_)_482_/F_625_ values corresponding to mismatched sequences are significantly lower than that of miR-92a-3p, indicating that mismatched sequences cannot give rise to efficient fluorescence recovery of Atto-425. According to the regression equation, the measured concentrations of SMT, TMT and NCT are 0.16, 0.15 and 0.13 pM, respectively, which are much lower than their actual concentrations. This indicates the proposed biosensor can selectively detect miR-92a-3p.

### 3.5. Detection of miR-92a-3p Extracted from Exosomes

**Figure 6:**
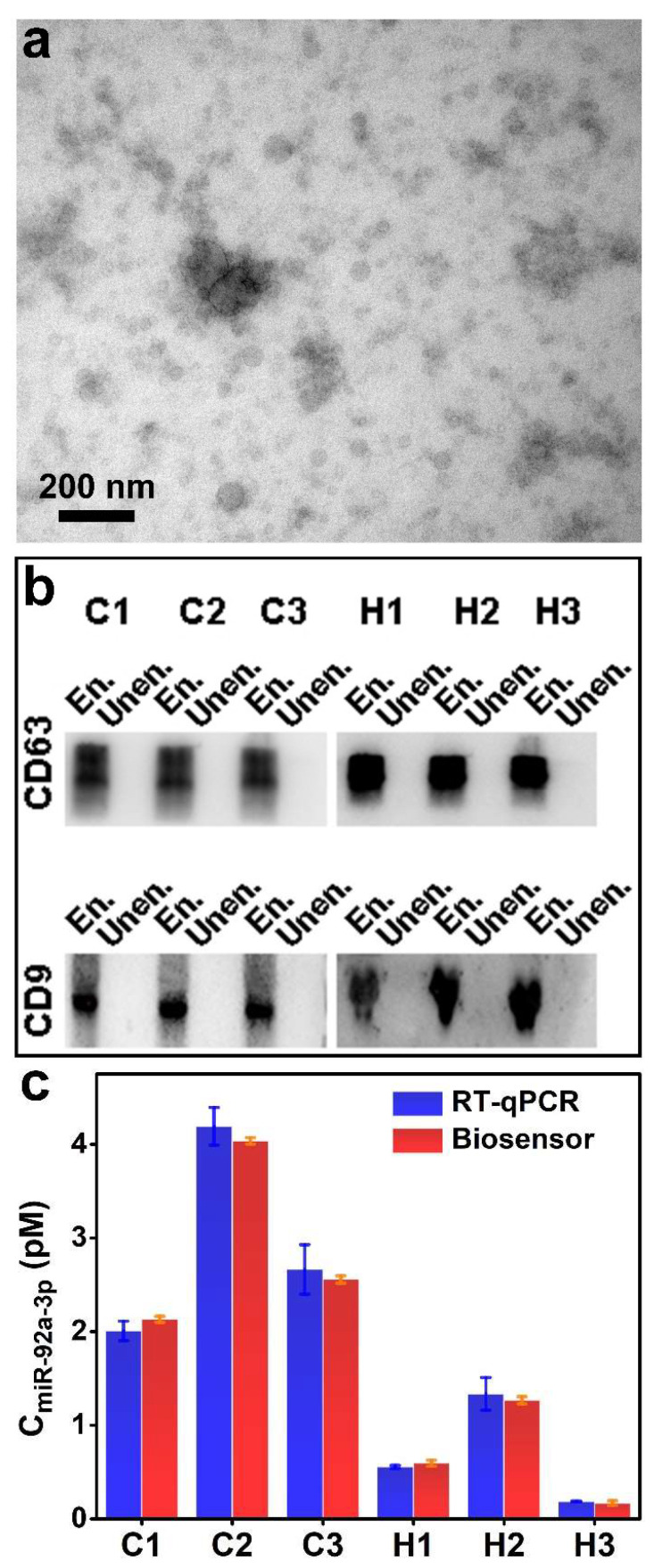
(**a**) TEM image of exosomes. (**b**) Western blotting of exosome-enriched (En.) and exosome-unenriched (Unen.) samples. C1~C3 represent CRC patients; H1~H3 represent healthy controls. (**c**) Comparison of the concentration of exosomal miR-92a-3p of three CRC patients and three healthy controls detected by RT-qPCR and the proposed biosensor (*n* = 3, mean ± s.d.).

Before performing the exosomal miR-92a-3p assay, exosomes were extracted from human serum, and their internal miR-92a-3p was obtained by cleaving the exosomes. At first, the morphology of exosomes was characterized by the TEM. They are spherical and exhibit dimensions in the range of 30–110 nm (Figure 6a). According to the literature, exosomes may shrink during TEM sample preparation, which results in the observed size of exosomes being smaller than their actual size [57]. Furthermore, exosomes extracted from three CRC patients and three healthy controls were identified by Western blotting to demonstrate their successful extraction. The marker proteins of exosomes, CD63 and CD9 were detected [58,59]. From Figure 6b, Western blotting images demonstrate that there are abundant CD63 and CD9 in exosomes. The significant binding in exosome-enriched samples validates the efficient extraction of exosomes.

To verify the accuracy of our fabricated biosensor, RT-qRCR and the ratiometric fluorescent biosensor were used in parallel to detect the concentration of miR-92a-3p extracted from exosomes. As shown in Figure 6c, the detection results of the ratiometric fluorescent biosensor are consistent with those of RT-qPCR, demonstrating that our fabricated biosensor possesses high accuracy and practicality. In addition, the detection results of the ratiometric fluorescent biosensor showed higher stability than RT-qPCR. Furthermore, due to the sequence modification flexibility of the probe, other RNAs can also be detected by this biosensor by simply changing the probe sequence.

## 4. Conclusions

In conclusion, a ratiometric fluorescent biosensor based on MWCNTs@Au NCs and DSN-assisted signal amplification was fabricated for the detection of CRC-associated miR-92a-3p extracted from exosomes. Because of the fluorescence reference capability of MWCNTs@Au NCs, this biosensor is stable enough to detect clinical samples. With the introduction of DSN-assisted signal amplification, this biosensor exhibits high sensitivity and a limit of detection as low as 31 fM. Furthermore, the specific cleavage characteristic of DSN endows this biosensor with high selectivity, even against single-base mismatched targets. Furthermore, because of the flexibility of sequence modification, this biosensor is able to detect other RNAs by simply changing the sequence of the probe. In view of these advantages, our fabricated ratiometric fluorescent biosensor shows the possibility of achieving early diagnosis for diseases by detecting miRNAs.

## Figures and Tables

**Table 1:** The oligonucleotide sequences used in this work.

Oligonucleotide	Sequence (5′ → 3′)
Probe	Atto-425-(CH_2_)_6_-ACAGGCCGGGACAAGTGCAATA
miR-92a-3p	UAUUGCACUUGUCCCGGCCUGU
Single base mismatched target (SMT)	UAUUCCACUUGUCCCGGCCUGU
Two base mismatched target (TMT)	UUUUCCACUUGUCCCGGCCUGU
Non-complementary target (NCT)	UGUCAGUUUGUCAAAUACCCCA

**Table 2:** Comparison of the detection performance of the ratiometric fluorescent biosensors and DSN signal amplification-based fluorescent biosensors for miRNAs.

Fluorescent Materials	Targets	Linear Interval (pM)	Limit of Detection (pM)	Ref.
DNA-AgNCs	miR-141	5 × 10^3^–1 × 10^5^	2.5 × 10^3^	[45]
Protonated phenyl-doped carbon nitride, ROX	miRNA-224	10^3^–2 × 10^4^	200	[46]
FAM, TAMRA	miRNA-21	10^2^–2 × 10^4^	73	[47]
NMM, DAPI	miRNA-21	10–4.5 × 10^4^	3.1	[48]
Chameleon Ag NCs	miR-17-5p	10–10^4^	2.8	[49]
CDs, FAM	miRNA-21	50–10^4^	1	[50]
CdTe QDs, FCMMs	let-7a	2–2 × 10^2^	0.1	[13]
Boron doped g-C_3_N_4_ nanosheets, Cu NCs	miR-582-3p	0.2–1	0.049	[25]
FAM	let-7a	0.5–5 × 10^2^	0.4	[51]
FAM	miRNA-21	0.1–1 × 10^3^	0.1	[52]
FAM	let-7b	0.5–10^3^	0.16	[53]
FAM	miRNA-141	5–10^3^	0.42	[54]
FAM	let-7a	0.1–2 × 10^3^	0.06	[55]
Hairpin structure molecular beacons	let-7a	1–10^4^	0.0325	[56]
MWCNTs@Au NCs, Atto-425	miR-92a-3p	0.1–10	0.031	This work

## Data Availability

Not applicable.
